# Breast Cancer Survivorship Programme: Follow-Up, Rehabilitation, Psychosocial Oncology Care. 1st Central-Eastern European Professional Consensus Statement on Breast Cancer

**DOI:** 10.3389/pore.2022.1610391

**Published:** 2022-06-02

**Authors:** Zsuzsanna Kahán, István Szántó, Rita Dudás, Zsuzsanna Kapitány, Mária Molnár, Zsuzsa Koncz, Mónika Mailáth

**Affiliations:** ^1^ Department of Oncotherapy, University of Szeged, Szeged, Hungary; ^2^ St. George’s General Teaching Hospital, Székesfehérvár, Hungary; ^3^ Department of Physiotherapy, Semmelweis University, Budapest, Hungary; ^4^ Oncoradiology Centre, Bács-Kiskun County Hospital, Kecskemét, Hungary; ^5^ Institute of Behavioural Sciences, Semmelweis University, Budapest, Hungary; ^6^ Institute of Oncology, University of Debrecen, Debrecen, Hungary

**Keywords:** follow-up, healthy lifestyle, physical rehabilitation, psychosocial oncology care, social rehabilitation, side-effect management

## Abstract

Follow-up includes ongoing contact with and health education of the patient, surveillance and control of the adverse effects of surgery, oncological therapies or radiotherapy, screening of metachronous cancers, and comprehensive (physical, psychological and social) patient rehabilitation, which may be enhanced by a healthy lifestyle. Primary attention should be paid to early detection and, when needed, curative treatment of local/regional tumour recurrences. Similarly, with the hope of curative solution, it is important to recognize the entity of a low-mass and relatively indolent recurrence or metastasis (oligometastasis); however, there is still no need to investigate distant metastases by routine diagnostic imaging or assess tumour markers. Below there is a list of possible sources of support, with respect to adjuvant hormone therapy continued during long-term care, social support resources, pivotal points and professional opportunities for physical and mental rehabilitation. Individual solutions for specific issues (breast cancer risk/genetic mutation, pregnancy) are provided by constantly widening options. Ideally, a complex breast cancer survivorship programme is practised by a specially trained expert supported by a cooperative team of oncologists, surgeons, breast radiologists, social workers, physiotherapists, psycho-oncologists and psychiatrists. The approach of follow-up should be comprehensive and holistic.

## Introduction

The recommendations below are based on the available literature in English and the authors’ own experience, and they are in line with comprehensive national and international recommendations on the topic published in English (1–3).

The document constitutes one of a series of guidelines developed by the consensus development panel method (4). Within a complex breast cancer survivorship programme follow-up care restricted to patients considered healed and various types of supportive and palliative measures that should start already at the diagnosis of breast cancer and should be practised throughout its management if needed will be reviewed.

Since all consensuses based on clinical practice and the current literature, this consensus will need to be updated as the field evolves. Panel members agree that as a future advancement, a dietitian, a self-help group leader and a GP expert will be involved in the update of this document.

## Follow-Up Care

Follow-up care means the regular check-up and support of breast cancer patients who are clinically tumour-free, usually have undergone breast surgery, and many of them need adjuvant hormone therapy (1–3). Follow-up care tasks:

• Communication with the patient, facilitating adherence to adjuvant treatment, coordination of care and rehabilitation.
• Health education, lifestyle advice (healthy diet, physical activity, etc.).
• Detection of relapse, rapid and effective assessment if relapse is suspected.
• Facilitating and supporting toleration of adjuvant hormone therapy.
• Detection, prevention and treatment of consequences of the disease and side-effects of surgical and adjuvant treatments (referral to mental, physical and social rehabilitation services, if needed).
• Tertiary screening: prevention and early detection of metachronous cancers (this is usually the same as the screening strategy for the average risk population; in individuals with BRCA mutations, breast screening, possibly gynaecological screening, and gynaecology assessment during tamoxifen therapy, annually or with individually determined frequency, is recommended).
• Declaration of the patient’s health status or need of treatments.
• Special aspects: genetic risk, pregnancy.

The atmosphere of long-term care differs from that in active oncology treatment facilities: patients should be empowered to return to their normal life and restore their health; they should be provided with help for full rehabilitation. Patients’ independence should be reassured, but at the same time they should be provided with a sense of security, support, and background for the disease they have overcome. During long-term care, the patients should receive adequate information about their situation, state of health and the procedures involved, so that they can fit it into their lifestyle; in the event of a relapse, quick and effective help should be provided to resolve the situation. All these require individualized, open communication, providing a sense of care, and an atmosphere of trust (4, 5). It may also be necessary to involve the patient’s family members and close relatives. Currently in Hungary, long-term care is provided by oncologists, but in many countries there is an effort to assign long-term care tasks to GPs or nurses. This requires training and protocols, as well as proper communication with the treatment team. Some of these tasks are highlighted below.

### Health Education, Lifestyle Advice (Diet, Physical Activity, etc.)

The most important aspect is making efforts to achieve healthy body weight, since primarily overweight, but also increased BMI have been associated with an unfavourable prognosis. Although the relation between cancer-related outcome and body weight or diet could not be demonstrated, these factors may adversely affect overall health (including anticancer therapy-related adverse effects), secondary cancer incidence and all-cause mortality rates. Optimal body weight is based on a healthy diet (high in fruits, vegetables, and whole grains and low in processed foods or added sugars) and a right amount of exercise that is not contraindicated even after breast surgery (see *Physical Rehabilitation*, also). It is recommended that patients stop drinking alcohol and quit smoking (1–3, 6, 7). For all these, thorough patient educational activity is needed or, in special cases the help of a registered dietitian.

### Detection of Relapse, Assessment of Suspected Relapse

When examining a patient, it is essential to keep in mind their individual risk for local/regional recurrence or metastasis. The risk of relapse depends not only on the primary tumour status, but also on the treatment administered. If the patient does not receive adjuvant therapy despite a high risk of recurrence, the vigilance of both the treating physician and the patient is essential, the latter being achieved by providing the patient with adequate information. Breast cancer subtype should also be considered: hormone receptor-negative and rapidly proliferating tumours may recur within 5 years after the first treatment, while the risk of relapse for hormone receptor-positive tumours remains constant for at least 10 years.

Long-term care is based on careful (purposeful) medical history and physical examination. Instrumental investigations for the assessment of systemic relapse (e.g., diagnostic imaging of the chest, abdomen, bones, tumour marker tests) are only required if there is an indicative complaint or symptom. Indeed, intensive assessment in asymptomatic cases will not affect either the time of diagnosis of metastasis or survival, but it may compromise quality of life due to anxiety and addiction. By contrast, diagnostic imaging of the operated breast and regional lymph nodes requires great care: after breast-conserving surgery, both the operated and contralateral breast should be assessed on a yearly basis, as recommended by a breast radiologist, usually via mammography and ultrasound or MRI (see the chapter on Breast Diagnostics) (1–3). For lobular carcinoma, it is particularly important that ultrasound scanning be part of a complex diagnostic imaging follow-up even after 5 years (3).

The diagnosis of an oligometastatic condition, which has been identified in recent years as a new biological entity, is of paramount importance (8). Radical local treatment of a slowly progressing and low-mass tumour can be life-saving in some cases. Therefore, if it is suspected, it should be rapidly confirmed with sensitive testing methods, with the hope of a curative treatment and favourable therapeutic outcome (9, 10).

In some cases (e.g., when they cannot present for long-term care due to a comorbidity), patients may be managed by a GP who would follow the recommended protocol. It is important to inform patients about the course of the follow-up care and the abnormalities that may occur due to the disease or the treatment.

### Detection, Prevention and Treatment of Consequences of the Disease and Side-effects of Surgical and Adjuvant Treatments (Support, Rehabilitation)

Expected side-effects and abnormalities depend on the type of treatment administered, the dose and duration of treatment, the patient’s age and comorbidities. Possible consequences of different treatments are shown in Table 1 (1–3, 11–19). Side-effects can lead to temporary or long-term decline in body image, physical condition and ability, and mental status, all of which will compromise quality of life (18).

**TABLE 1:** Adverse consequences of breast cancer treatments complained during follow-up.

Treatment	Side effects developing during treatment	Side effects developing months or years after treatment
Surgery	Numbness, body image problems, cosmetic outcome, sexual dysfunction, restricted motion of the shoulders, pain	Lymphoedema, neuropathy, restricted motion of the shoulders, pain
Radiotherapy	Skin lesions, breast fibrosis, asymmetry, cosmetic issues, pain, body image disorders, sexual dysfunction, pneumonitis, lymphoedema	Soft tissue fibrosis, ischaemic heart disease, radiogenic secondary malignancy
Chemotherapy	Cognitive impairment (“chemo brain”), fatigue, early menopause, infertility, sexual dysfunction, hair loss, weight changes, neuropathy, cardiomyopathy	Sterility / hormone deficiency / menopause, osteoporosis / osteopenia, leukaemia / myelodysplastic syndrome, cardiomyopathy
Trastuzumab	Reversible heart damage	
Tamoxifen	Hot flushes, menstrual disorders, mood disorders, vaginal discharge/infection, elevated triglyceride levels	Stroke, endometrial cancer, thromboembolic event, osteopenia in premenopause
Aromatase inhibitors	Vaginal dryness, decreased libido, joint and muscle pain, increased cholesterol levels, gastrointestinal symptoms, urinary incontinence, impaired cognitive functions	Osteoporosis, risk of fracture

Due to changes in body image, various tools (wigs, breast prostheses, etc.) and breast reconstruction may be considered as immediate or delayed solutions. Complex treatment of the issue is recommended (physical and mental help).

Lymphoedema should be prevented by losing weight if the patient is overweighted and by protecting the arm (physical activity is allowed, but weight-bearing by the arm should be avoided, efforts should be made to prevent erysipelas, but venous access to the arm or blood pressure measurement on the operated side is not contraindicated, moreover it may even cause anxiety if it were prohibited (14).

Monitoring of cardiotoxic consequences should be continuous during active oncology treatment; during long-term care, special cardio-oncology care is needed for patients at risk (pre-existing heart disease, prior oncological treatment with cardiotoxic drugs or cardiac/coronary artery radiation exposure), or if there are symptoms suggesting cardiac disease (breathlessness, fatigue, cardiac decompensation) (15,16).

Monitoring bone health and osteoporosis should depend on age and the treatments administered. In case of chemotherapy-induced menopause or endocrine therapy, a baseline DEXA test should be performed and then monitored depending on the treatment (Table 2). For joint complaints, rheumatology examination is recommended and physiotherapy may be deliberately used (11). For musculoskeletal complaints caused by aromatase inhibitors, switching to tamoxifen or another aromatase inhibitor may be the solution, if necessary.

**TABLE 2:** Follow-up assessments during adjuvant endocrine therapy.

Medication	Premenopause	Menopause
Tamoxifen	DEXA every 2–3 years Yearly gynecology checkup	Yearly gynecology checkup
GnRH/LHRH analogs	DEXA	—
Aromatase inhibitors	DEXA every two years	DEXA every two years

GnRH, gonadotropin releasing-hormone; LHRH, luteinising hormone-releasing hormone; DEXA, Dual Energy X-ray Absorptiometry (bone density measurement).

Fatigue, mental disorders and cognitive impairment are well-demonstrated as a consequence of chemotherapy, but not fully clarified in the case of hormone therapies (17–21). During long-term care, it is worthy gathering information on this issue and initiating the patient’s rehabilitation, if needed.

The use of a lubricating cream or suppository in case of sexual complaints or vaginal dryness may be tried, and medicinal treatment or pelvic floor exercises may be recommended for urinary incontinence (22).

### Managing Endocrine Therapy

Adjuvant hormone therapy is usually recommended for a period of 5–10 years, but due to its long duration and successful return of the patient to a normal life, and partly due to possible side-effects, medication adherence is poor in a significant proportion (up to half, according to certain estimates) of patients. Therefore, one of the most important goals of long-term care is to promote good therapy adherence. Ensuring that patients are informed and perform appropriate follow-up tests, as well as side-effect management, will improve results. Table 2 shows the recommended follow-up assessments for various treatments. Either due to chemotherapy-induced amenorrhoea or due to GnRH analogues, menopausal symptoms may develop in the form of hot flushes, mental instability, sexual complaints (decreased libido, vaginal dryness), which are deteriorated by aromatase inhibitors (22, 23). Aromatase inhibitors may cause androgen-type alopecia, too. Tamoxifen is more likely to induce vaginal discharge and weight gain. Gabapentin, a selective serotonin reuptake enhancer (SSRE) and lifestyle changes may help in reducing hot flushes, while topical treatment may be considered to help sexual complaints, e.g. lubricant, vaginal suppositories, or laser treatment, as a novel opportunity (1–3, 24). Hormone replacement therapy, even the use of oestrogen-containing vaginal creams, is contraindicated. Rheumatological treatments can be administered for joint or muscle pain (especially common with aromatase inhibitors).

### Special Aspects: Genetic Risk, Pregnancy

When a hereditary predisposition to breast cancer is suspected, great caution and tactfulness is required and a sufficiently long time should be allowed for processing the informations (25–27). In cases of a family history suggesting inherited risk of cancer, cancers at a young age, or specific tumour types, testing for BRCA or other hereditary gene mutations is essential and recommended by numerous international guidelines. If justified, and the patient is ready to accept it, the patient may be referred to a genetic counselling centre; ideally this is done at the time of the initial care. If a pathological gene mutation carrier status is confirmed, this has a number of consequences for the follow-up care: preventive breast surgery or adnexectomy depending on future family plans (the risk-reducing effect of Fallopian tube removal with preserving the ovaries is being evaluated in a clinical trial), developing a specific breast screening strategy if needed, or other actions may be considered based on the advice of a geneticist; naturally, the issue of informing and screening the family members also arises.

The issue of undertaking pregnancy depends on the risk of relapse, how this changes over time, and the nature and timing of the administered treatments. During the discussion, it is worthy to understand whether the patient sees her illness in a realistic way and, if necessary, to provide objective information about the situation. There is no evidence that pregnancy *per se* would be detrimental in terms of recovery or recurrence. Chemotherapy may lead to infertility for a shorter or longer period of time; one of the reasons is that hormone production is impaired, although this risk can be reduced by using a GnRH analogue during chemotherapy. The ability to regenerate after chemotherapy and the chance for recovery of fertility decrease with age (28). For infertility, the patient should be referred to a specialist. Due to the genotoxic effects of chemotherapy, a waiting period of at least 3 years is required after chemotherapy. For a successful pregnancy, hormone therapies should be terminated; if the patient received tamoxifen, a latency of 3 months is required before pregnancy, due to the slow clearance of the drug.

## Rehabilitation—With a Holistic Approach

Note the general and official WHO definition for rehabilitation (1980): “Rehabilitation is an organized assistance needed by people with a long-term or permanent damage to their health, physical and/or mental integrity in order to reintegrate into society and their communities. A coordinated, individualized set of medical, pedagogical, social and occupational measures aimed at making the rehabilitated individual a happy and, if possible, a full-fledged citizen of the society. Rehabilitation is a social task.”

The original meaning of the word rehabilitation is good news, the restoration of lost honour, satisfaction—within this conceptual framework, the physician or the caring community should assist in restoring the patient’s self-esteem and reduce the losses associated with illness (29, 30).

The rehabilitation of a breast cancer patient begins at the time of diagnosis, no matter whether it is an operable/early stage case and has received curative treatment(s), or advanced or metastatic breast cancer that requires continuous treatment and intensive monitoring. Rehabilitation is comprehensive (physical, mental, social) and is conceptually planned; not an *ad hoc* process. Naturally, rehabilitation is tailored to the prognosis of the disease, which can be estimated based on prognostic factors. Altered physical condition and the presence of mental problems are well known issues, and when these appear and are recognized, it is the oncologist’s responsibility to refer the patient to a specialist in the appropriate field (physiotherapy, reconstructive surgery, psychosocial oncology care, social worker, etc.). During the follow-up period, the task of the oncologist is to prevent and recognize the symptoms and to refer the patient to an appropriate specialist. For rehabilitation purposes, it would be essential to avoid the stigma of the disease and, while underlining the importance of the investigations, treatments and follow-up, it should be ensured that the disease did not become a central issue of the patient’s life, or a determinant of all goals and activities. Comprehensive life counselling is the task of the oncologist that helps the patient’s reintegration into the community of the healthy. For effective rehabilitation, it is important to set realistic goals and to take into account the patient’s individual physical and mental condition and psychointegrative harmony. A prerequisite for effective rehabilitation is that specialists in the physical, mental and social spheres, working as a team, are available when necessary, and provide assistance in all aspects of rehabilitation. Within a comprehensive breast cancer survivorship programme various forms of rehabilitation are usually provided at the initiative of the staff who provides care, treatment or follow-up for the patient (29, 30).

The important role of patient advocacy and primary care in the holistic approach should be also emphasized. In fact, breast cancer was the first example for initiating patient advocate activity, and Europa Donna was the first breast cancer advocate group that established a Europe-wide coalition (31). In most countries there are various self-established patient groups that not only provide direct support to patients and their families, but raise social attention, public awareness, reduce stigmatisation and, may have impact on politics too. General practitioners may overtake many breast cancer-specific tasks depending on the need or actual situation such as providing certain tests or delivering certain medications, diagnosing or controlling comorbidities sometimes related to cancer therapy itself, or guiding life style changes etc. In both fields the most important aspect and need is the maintenance of ongoing communication, contact and mutual confidence between the members of the patient advocate group/primary care physician and the representatives of the cancer multidisciplinary expert team.

## Social Rehabilitation

### Oncology Social Work

Social work is a supporting activity classified as an applied social science, which promotes social development, improvement of functioning and solving issues at the individual, group and community levels. Hospital social work helps to solve the patients’ and their families’ social issues. Support can also be requested from the Family Support Institute of the Local Government. Social workers’ tasks may include supporting the achievement of social and financial security, mediating individual social services, helping patients back to their home, or guiding patients toward psychosocial oncology care when mood disorders and anxiety are recognized.

### Supporting the Social Rehabilitation of Breast Cancer Patients

Social rehabilitation means the process of integration into the community, the criteria of which are the existence of social relationships, relative financial and economic autonomy and the ability to ensure the means of subsistence. Social rehabilitation begins from the moment the diagnosis is established, and continues throughout the treatment period and sometimes the follow-up care period.

Breast cancer is an oncological disease that primarily affects women. The traditional family model of our society has changed, with every second marriage ending in divorce. In many cases, women are breadwinners, and in 86% of single-parent families, it is the mother who raises her children alone. People living in traditional families are also characterized by a “dual-earner” model, so that if the wife/mother falls ill, the family loses earnings (32). This disease brings changes in the lives of those affected and their relatives, and family members need to adapt to this and promote adaptation in others. Limitations of mental and physical stress tolerance, social disadvantages and lack of resources must also be taken into account.

### Most Common Social Issues and Their Solutions

In the presence of an oncological disease, patients often cannot keep their jobs due to the treatment, side-effects, and mental strain. It is essential that patients/clients themselves decide whether they feel physically and mentally capable to continue their work (33, 34). If they are unable to perform their job on a permanent basis, they may claim insurance and social benefits to compensate the loss of earnings.

We have included the forms of institutionalized social support in Hungary as an illustration in Supplementary Appendix S1.

Recognition of the psychological processes and reactions and of depression and anxiety symptoms associated with oncological diseases and treatments contributes to the establishment of patient/client compliance skills and that of a good doctor-patient relationship. The patient’s/client’s personality and potential coping mechanisms should be taken into account. These are influenced by the patient’s values, socialization, attitudes, stress management skills, and also by social factors, workplace and family environment, and whether the patient/client has mental illness or addictions. If depression and anxiety disorders exist or develop, or in the event of need of crisis intervention, the patient/client should be referred to a psychiatrist or psychologist. The patient’s/client’s mental condition should be monitored since the time of diagnosis, and the help of a specialist should be sought if any change occurs or if a period of the illness may lead to mental vulnerability. It is important that the patient’s/client’s attitude to mental health would allow the acceptance of the psychological support needed for recovery. Coping with the disease is aided by avoiding isolation and sustaining family, friend, and community relationships. Patients/clients should be guided toward self-help groups and patient organizations, in which they will have the opportunity to share their problems with peers dealing with similar illnesses, who reach out with understanding and set an example of positive vision. After recovery, successful rehabilitation will result in the patient being employed and self-sufficient, which is enabled through employment rehabilitation. Employment rehabilitation means that a previously employed person, who currently has altered work capacity due to illness, is employed in a job matching her current working aptitude. Useful work provides the patient/client with an opportunity to restore self-fulfilment, self-esteem and a sense of worth.

## Physical Rehabilitation

### Introduction

According to a WHO survey, sedentary lifestyle is the fourth most important risk factor for current endemic diseases worldwide, including cancer. Physical activity means exercise associated with any muscle contraction involving a change in location or position that requires a higher energy expenditure than at resting level. Isometric and isotonic, eccentric and concentric muscle work can be part of physical activity. Established physiotherapy is an essential part of the complex management of breast cancer all along the disease continuum; since no other chapters of this series deal with physiotherapy, here we summarize the related aspects irrespective of the phase of the disease.

As a result of regular exercise, the organism undergoes structural, functional, and physiological changes that help to prevent and delay many diseases, or recover from them. This effect is also influenced by the form, intensity, duration, and timing of the exercise. To measure the magnitude of the load, we use the term “metabolic equivalent of task (MET),” which is based on measuring oxygen consumption. Knowing the MET value of physical activities, a desired weekly load can be easily established (Table 3). Based on the WHO proposal, American and European exercise recommendations were formulated for healthy individuals (Table 4).

**TABLE 3:** Approximate energy expenditures for selected forms of activities.

Category	Self care	Occupational	Sport	Physical conditioning
Very light MET 3	Bathing, shaving, dish washing, dressing, writing, driving, desk work	Sitting (office) or standing (service) work, truck driving, operating a crane	Playing billiards golf, archery, boating, slow dancing	Walking at 3 km/h, stationary exercise bike with very low resistance, very light gymnastics
Light MET 3–5	Window cleaning, leaf-raking, weeding, sickling, machine mowing, painting, carrying items weighing 7–15 kg	Shelving light objects, light welding, light carpentry, repairing machines, car fixing, hanging pictures, wallpapering	Dancing, golf (walking), sailing, volleyball, doubles tennis, horse riding	Walking at 4.5–6 km/h, cycling at 9–12 km/h, light gymnastics
Moderate MET 5–7	Easy digging, hand grass levelling, slow stair climbing, carrying loads weighing 15–30 kg	Easy carpentry, garbage shovelling, use of pneumatic tools	Badminton, singles tennis, skiing (downhill), light backpacking, basketball, football, ice skating, galloping	Walking at 6.5–7.5 km/h, cycling 9–12 km/h, swimming (breaststroke)
Difficult	Wood sawing, heavy shovelling, stair climbing at limited speeds, carrying loads weighing 30–45 kg	Firing in a furnace, trench digging, pickaxing, shovelling	Canoeing, playing rugby, mountaineering, fencing	Jogging, swimming (freestyle), cycling at 18 km/h, heavy gymnastics, rowing machine workout
MET 7–9				
Very difficult MET 9	Carrying load on stairs, carrying loads over 45 kg, fast stair climbing, heavy snow shovelling	Wood cutting, hard physical work	Handball, squash, skiing (hiking), intense basketball playing	Running at > 9 km/h, cycling at > 18 km/h or uphill, rope jumping

MET, metabolic equivalent of task.

**TABLE 4:** Minimum recommended exercise for healthy individuals.

American recommendations	European recommendations
at least 150 minutes/week of moderate intensity or 75 minutes/week of intense aerobic exercise	Minimum 30 minutes of moderate-intensity exercise 5 days a week or at least 20 minutes of vigorous exercise 3 days a week
Exercise should consist of units lasting at least 10 minutes	Activity can be gathered from units of at least 10 minutes
Further beneficial effects result from increasing workout time to 300 minutes/week for moderate-intensity or to 150 minutes/week for vigorous aerobic exercise, in adults. It is recommended to perform moderate or high intensity muscle strengthening activity for 2 or more days, involving all major muscle groups	It is recommended to perform additional muscle strengthening and endurance exercises 2–3 days a week

### Physiological Effects of Physical Exercise

• Exercise activates natural killer cells (NK cells) that play a role in killing cancer cells.
• It reduces the body’s susceptibility to bacterial infections.
• Supports body weight control.
• Prevents deterioration of cardiorespiratory endurance, which may occur as a side-effect of cardiotoxic antitumour therapies.
• Helps to recover muscle mass, reduces sarcopenia due to disease and treatments.
• Reduces the risk of thromboembolic complications, the incidence of which is 7-fold higher in cancer patients than in the average population.
• Supports correction of abnormal movement patterns, develops the ability to coordinate and maintain balance, which is deteriorated as a common consequence of polyneuropathy caused by chemotherapy.
• Reduces fatigue.
• Reduces symptoms of musculoskeletal syndrome causing bone, muscle, and joint pain and stiffness.
• Increases bone mineral content, which is important for bone loss due to hormone and chemotherapy, and thus reduces the risk of bone fractures.
• Improves self-esteem, reduces the effects of distress, anxiety, fear, pain, and initiates positive self-healing processes.
• Reduces the decline of cognitive functions and slows down the ageing process.
• Reduces the risk of developing lymphoedema.

### Workout Forms

Aerobic or cardio-training is a continuous or intermittent intense workout of the large skeletal muscle groups for 20–50 min. This type of exercise primarily improves endurance and increases the capacity of the cardiorespiratory system. It includes walking, Nordic walking, running, swimming, cycling, stair climbing, ball sports, etc.

Anaerobic or resistance training is a short-term high level effort that helps to prevent muscle atrophy and osteoporosis. Typical forms of resistance training are weightlifting or sprinting.

Other exercise types, such as breathing gymnastics, proprioceptive training, stretching, etc. can be incorporated into both training types. Different exercise types are not interchangeable, it is the task of a physiotherapist to set an individualized training programme.

The physiotherapist can find out the patient’s usual physical activity or fitness *via* a specific questionnaire, such as the IPAQ (International Physical Activity Questionnaire), and can create an individual training plan for the patient based on the FITTA criteria: frequency, intensity, time, type of the exercise and perseverance (approach), and the 5R criteria: Repetitions, Rate, Range, Resistance, and Rest (Table 5).

**TABLE 5:** Options of functional locomotory tests.

Function, abnormality	Tool	Manual examination by a physiotherapist
Range of motion (ROM)	Goniometer	functional tests
Muscular strength	Dynamometer	Oxford scale (0–5)
Upper limb volume	optoelectric instrument plethysmography water displacement method Khunke’s volume formula	a state characteristic (Khunke’s formula) recorded on the basis of a series of circumferences (k1, k2…) measured every 4 cm perpendicularly to the axis of the affected limb, suitable for follow-up $\sum V=\frac{K_{1}^{2}+K_{2}^{2}+K_{3}^{2}+K_{n}^{2}}{\Pi }$
Scarring, axillary web syndrome, AWS		visible and / or palpable cording pain restricted ROM for flexion and abduction (usually an axillary phenomenon, but elbow and wrist involvement may also occur)

### The Place of Physiotherapy in the Perioperative Care of a Breast Cancer Patient

Breast cancer therapy most often begins with surgery, so it is recommended that the physiotherapist be in touch with the oncology team, so that they will be informed about the type of surgery and have the opportunity to meet the patient. It is important that the physiotherapist has a BSc or MSc degree, experience in the field of oncology and a close professional relationship with the surgeon and oncologist (35).

Both the period of preparing the patient for surgery and the early postoperative period impose tasks on the physiotherapist and at the same time affect the patient’s later quality of life and the outcome of the disease (36). Early mobilization and physiotherapy will significantly reduce the functional impairment caused by the disease and interventions.

Complex functional impairment of the upper extremities associated with breast surgery may develop including the following:

• Pain, hyperaesthesia, paraesthesia,
• Stiffness,
• Secondary lymphoedema,
• Seroma,
• Scarring (axillary web syndrome, AWS),
• Decreased muscle strength and restricted motion, limited range of motion (ROM),
• Weakening of grip strength of the hand,
• Complex functional impairments,
• Decrease in daily activity,
• Sensory disturbances/losses in the chest area,
• Posture/body image disorder,
• Neck/shoulder girdle dysfunction (involvement of the upper part of the trapezius muscle) (37).

Early and late functional complications of breast cancer treatment along with patient quality of life have long been studied, and a variety of methods are available to manage these in routine patient care (Table 5). The possibilities for prevention and treatment will be discussed after a presentation of methodology. Assessing both the range of motion of the shoulder and muscle strength of the upper limb is important. Decrease in grip strength of the hand and a limited range of motion pose serious problems to the patient. Both functional tests and other measuring tools can be used to assess functional restriction, which is also a prognostic indicator (38).

Measurement of the upper limb volume can be performed using several methods, and this will significantly help in the early detection of lymphoedema. Circumference differences measured at six anatomical points are highly correlated with the results of water displacement volume measurement (39).

AWS caused by scarring is a typical group of signs and symptoms following oncological breast surgery. In most of the cases, a scarred cord-like lesion is palpable in the armpit; in a milder form it is only perceived by the patient, and therefore recording subjective symptoms is essential. Predisposing factors, incidence, pathological aspects, and therapeutic options for AWS are being actively researched. The lesion usually develops in the armpit, but it may extend down along the elbow pit to the base of the thumb. The syndrome is caused by the occlusion, inflammation and later on the fibrosis of the superficial lymphatic vessels, as a consequence of surgery (40).

The current trend is the global analysis of upper extremity functions that is in addition to the measurement of the range of shoulder motion and anatomical parameters of the upper limb, complex upper limb functions needed to perform everyday tasks, as well as circulatory conditions and physical stress tolerance are assessed (41–45). Questionnaires completed by the patient are also included (Table 6).

**TABLE 6:** Questionnaires designed for complex examination of upper limb functions in patients with locomotor disorders.

“The Disabilities of the Arm, Shoulder and Hand”, **DASH**	To measure complex functions of the upper limb	30 questions, of which 25 ask about functions related to lifestyle, and 5 about other symptoms (score 1–5) optional questions related to work, sports, artistic activities (4 for each category)	high score weak function
10 minutes (42, 43)			
**QUICK DASH**	An abbreviated version of DASH can be evaluated if there are >9 responses	11 questions	high score poor function
3 minutes			
“Upper Extremity Functional Index”, **UEFI**			
3 to 4 minutes (44)	To measure upper limb function	20 questions (score 0–4)	high score good function
**“Functional Assessment of Cancer Therapy”, FACT-B** 10 minutes (45, 46)	Multidimensional quality of life questionnaire	36 questions (score 0–4)	high score good quality of life
**FACT-B+4** 10 minutes (45, 46)	Multidimensional quality of life questionnaire expanded with questions on 4 upper limb functions	40 questions (score 0–4)	high score good quality of life
“Kwan’s Arm Problem Scale”, **KAPS** 3 to 5 minutes (40)	Upper limb function questionnaire for cancer patients	13 questions (score 1–5) it is also a psychometric indicator pain, stiffness, swelling, function	high score with more symptoms and poor function
“Subjective Perception of Post-Operative Functional Impairment of the Arm”, **SPOFIA** 3 minutes	To assess condition after breast cancer surgery	15 questions swelling, pain, anaesthesia, restricted range of motion and decreased muscle strength	a high score indicates marked upper limb damage

#### Preparing the Patient for Surgery

• Assessment of structural and functional condition using the aforementioned tests.
• Evaluation of comorbidities.
• Teaching early mobilization exercises.
• Thrombosis prophylaxis and teaching patients venous exercise and how to use compression bandages.
• Information on the symptoms and prevention of occasional lymphoedema.
• Assessing the need for and use of an aid (optimal prosthesis, bandage etc.).
• Explaining the role of exercise and physical activity in the healing and rehabilitation process.

#### Early Postoperative Tasks

• Positioning depending on the type of surgery.
• Early mobilization; the goal is to reach a vertical position as soon as possible (sitting, standing, walking).
• Early breathing exercise, chest mobilization to help prevent respiratory complications.
• Vascular physiotherapy or an elastic bandage or anti-thrombosis stocking applied before mobilization reduces the risk of thrombosis.
• Passive, assisted and then active movement of the upper limb on the affected side, teaching facilitation possibilities.
• Prevention of contractures.
• Core stabilization and mobilization.
• Restoring abnormal muscle balance caused by an altered body image.
• Preparing for a complex exercise programme, enrolling the patient in a small group class, as soon as possible.
• After reconstructive surgery (TRAM, LD, DIEP), lifting the arm above 90° have to be avoided for 3–5 weeks.
• Recovery of self-sufficiency functions (measurement of independence based on physical and cognitive capacity according to the “Functional Independence Measure, FIM” scale).

This period lasts for a couple of days, but in case of breast reconstruction surgery it may take longer time. Prior to hospital discharge, patients should be enrolled in a rehabilitation support group, when possible, in which they participate in a regular exercise programme under the guidance of a specialist, preferably a physiotherapist. If this is not available, an exercise programme should be created, which can be performed independently by patients in their home, and sports and other leisure time activities may also be suggested. Since oncology treatments after surgery (radiation and/or chemotherapy, hormone therapy, etc.) are also very demanding on the body, regular physical activity and exercise are essential.

### Lymphoedema

Although over the last decade, the widespread adoption of sentinel lymph node biopsy and patient training have significantly reduced the development of upper limb lymphoedema, it is essential that all lymph node-positive breast cancer patients who have undergone surgery, chemotherapy, or radiation therapy are considered potential lymphoedema patients. Therefore, all interventions and physiotherapy procedures causing significant hyperaemia of the affected upper limb should be avoided. (Harmful effects of blood pressure measurement, blood sampling or possibly intravenous treatment have not been confirmed, but are rather an assumption; regrettably, unjustified fear may cause anxiety in the patient.) Patient information, regular movement therapy, and manual lymph drainage (MLD), if needed, all support the functioning of the lymphatic system possibly damaged by the various oncological interventions, and reduce the probability of the progression of the lymphoedema. Because MLD stimulates lymphatic system activity, treatment should only be initiated with the recommendation of the oncology team since it may even pose a risk to the patient. Lymphatic drainage can be performed by a physiotherapist with specialist knowledge of lymphatic drainage in the field of oncology (46).

Complex lymphatic therapy also includes compression treatment, which may use bandages, stockings, and mechanical compression. It is important to know that use of these measures is not optional.

#### Compression Elastic Bandage

• Short-elongation, high working pressure elastic bandages are used.
• Applied in multiple layers with pressure decreasing evenly from distally to proximal direction (100%–70%).
• After manual treatment, it should be applied and maintained while the patient is performing active muscle activity.
• This is repeated daily until the reversible mobile part of lymphoedema is removed.

#### Compression Stockings

• Can be used at 1 to 3 compression gradients.
• Its purpose is to maintain an oedema-free state.
• In some cases it can also be used for preventive purposes.
• The type, size and gradient of stockings should be determined together with the attending physician.
• The stage of lymphoedema and the general condition of the patient and possible comorbidities should also be taken into account.

#### Machine Compression

• A complementary procedure, it must not be used alone without other anti-oedema therapies.

Early mobilization and active exercise programmes (30–50 min three times per week), complemented with MLD therapy, may significantly reduce the development and progression of the lymphoedema.

Complete decongestive therapy (CDT), which includes both MLD and compression therapy, significantly reduces pain and feeling heaviness in the arm (47).

### Conclusion

With their multiple beneficial effects, regular physical activity, sports and leisure activities improve quality of life and life prospects after complex breast cancer treatment. Due to the effects of complex treatment, age-specific characteristics and comorbidities, many of the patients do not know what type of exercise they may or should perform; the help of a physiotherapist is essential. Physiotherapists participate in the complex breast cancer survivorship programme in cooperation with the other specialists, their specific task and responsibility is building, teaching and supervising short-term and long-term exercise programmes. Physiotherapists may be involved in supporting breast cancer patients at the clinic, specialist care, primary care, home care service and in patient organizations all along the disease course according to the actual situation and need. Physiotherapy exercises and other forms of physiotherapy are now a part of integrative oncology and modern comprehensive breast cancer therapy.

## Psychosocial Long-Term Care and Rehabilitation

### General Guidelines for Psychosocial Oncology Care

It is now worldwide accepted that psychosocial care and psychosocial rehabilitation of patients diagnosed with breast cancer should be provided as an integral part of complex oncology care (48). This should begin when the diagnosis is communicated to the patient, and be practised within a complex cancer survivorship programme later on.

Relevant recommendations are summarized below, and these explain specific features of care based on general guidelines in psychosocial oncology care (49) and a recent protocol published by the Hungarian Ministry of Health (50). The summary is intended for all the psychologists, clinical psychologists, psychotherapists, psychiatrists, social workers, mental health professionals who work at an oncology centre providing active medical treatment, at an oncology department/outpatient clinic, at a crisis centre for cancer patients and their relatives or in private practice.

Interventions should be adapted to the oncology treatments being given and the patient’s current condition, and therefore close collaboration is required between the attending physician and the professional providing psychosocial care, who ideally is a member of the multidisciplinary team (1, 13, 18, 48, 51–63).

A person diagnosed with breast cancer may need psychosocial support and treatment throughout the entire course of the disease (Table 7).

**TABLE 7:** Common psychosocial symptoms that occur during certain stages of the disease.

Stage of the disease	Possible psychological / psychiatric phenomena and symptoms
Secondary prevention/cancer screening	Anxiety, communication and compliance difficulties, fear of social stigmatization, health anxiety, negligence, fear caused by a positive family history, procrastinating behaviour
	Psychosocial consequences of confirmed high genetic risk (e.g. insecurity, anxiety disorders, fear of disease)
Diagnostic work-up	Establishment of a doctor-patient relationship and its difficulties; the patient is becoming “familiar” with the health care system, the patient’s early experiences are “engraved” and will be decisive; the impact of issues related to the health system on the patient.
	Fear of “violation” of bodily integrity, fear of pain, fear of the patient role, fear of the loss of autonomy. Temporary narrowing of concentration and thought processes. Frequent intrusion into the private sphere (a matter of trust and attachment!), depersonalization, loss of security, chronic stress (long waiting times, fear of illness)
Communication of diagnosis, preparing for surgical procedures, discussing the treatment	A diagnosis of cancer may often induce psychological trauma, a mental crisis. In addition to the most common fears raised when the diagnosis is communicated (fear of death, loss of autonomy, pain, treatments, etc.), anxiety and depressive disorders (e.g., PTSD), cognitive dysfunction (e.g., restricted thinking and focus of attention), topic-specific problems should be highlighted: body scheme changes, self-esteem, partnership and sexual issues.
	When a patient first finds out the diagnosis, there may be violent emotional reactions, extreme manifestations, and complete introversion may even occur, which are natural emotional reactions to shock; however, they may require crisis intervention.
	Information and preparation before the (new) oncotherapy phase reduce anxiety and improve compliance.
Oncotherapy (surgery, chemotherapy, radiation therapy, hormone therapy)	Increasing communication difficulties (between patients, physicians, the medical staff and the relatives of the patient) due to physical and mental stress.
	Frustration, adjustment difficulties, mental regression, fear of death, internal / external body image disorders, depressive symptoms (due to the loss of health, but may also be biologically or drug-induced or of CNS origin), anxiety, psychosomatic symptoms, PTSD, relationship and sexual problems, psychogenic side effects.
	Early side effects of chemotherapy, anticipatory nausea and vomiting may lead to treatment discontinuation and prolonged aversion, reducing the possibility of re-treatment in the event of relapse.
	Unrealistic adherence to or rejection of treatment.
	Cognitive impairment after chemotherapy: impairment of concentration and integration, learning disabilities (20%–50%), mild decrease in IQ (cognitive impairment may be exacerbated by psychogenic factors).
	In patients with non-cerebral metastases, mild EEG abnormalities, paraesthesias occur in about 20% of cases. Changes in sexual life, family task allocation, relationship problems.
	Increasing financial burdens may change the patient’s economic and social status.
	Elevated levels of distress (sleep disturbance, restlessness, mood swings, anxiety, depressed mood, depression, fatigue syndrome), which compromise quality of life.
	As a result of regular or long-term hospital treatments, hospitalization, separation from the family and social isolation may develop.
	As a result of increased physical and mental strain, premorbid psychiatric problems may become exacerbated or decompensated; therefore, special attention should be paid to people who have been previously diagnosed or have avoided psychiatry, but are currently suffering from some form of comorbid psychiatric disorder (the importance of screening!).
	Any treatment type may cause anticipatory anxiety symptoms, grief reactions (due to loss of health or independence, etc.), and anticipatory bereavement.
Follow-up phase/ relapse-free phase	Adaptation difficulties, persistence of conditioned psychogenic side-effects, cognitive impairment, chronic fatigue, Damocles’ syndrome, PTSD, sexual disorders, development and exacerbation of addictions; loss of security, psychosomatic symptoms, mood disorders (depression), anxiety disorders (panic disorder, hypochondria, carcinophobia), risk of suicide.
Relapse, palliative care	Emotional crisis, anger, anxiety, depression, fear of death, adjustment / coping difficulties. Increased guilt, emotional instability, tension, anger, overt or hidden hostility, intellectual inhibition, mental regression, depersonalization.
Terminal stage	Fear of death, anxiety; rejection (denial), anger, bargaining, depression, resignation.

#### The Main Crisis Points May Include

• The period of assessment for the suspected disease.
• Establishment of the diagnosis.
• Preparing for surgery, starting oncological therapy.
• Initiation of oncological therapy, facing the burdens and side-effects of treatment.
• Follow-up/relapse-free period, “recovery to life.”
• Relapse, appearancediagnosis of metastases.
• Terminal stage.

#### Important Psychosocial Changes Following the Diagnosis of Cancer

• Emergence of fear of death, dealing with the issue of financial difficulties.
• Changes in body scheme that cause identity confusion (in terms of femininity, motherhood).
• Partnership and sexual problems.
• Difficulties of lifestyle change.
• Financial problems.
• Unbalanced family homeostasis, reversal of roles.
• Uncertainty about the future.
• Fear of recurrence of the disease.

#### Interventions That Can Be Used Effectively in Mental Care

• Psychoeducation.
• Crisis intervention.
• Psychological counselling.
• Supportive-expressive psychotherapy.
• MBCR (mindfulness-based cancer recovery) programme.
• Relaxation, autogenic training, “imaginative” therapies.
• Other individual and/or group therapeutic techniques, depending on the qualification and skills of the professional providing the care.

For all these, it is essential:

• To assess and be aware of the patient’s physical/mental condition (tumour stage, histological type, age, presence of risk factors, level of social support, living conditions, premorbid personality, comorbidities, previous life events, etc.).
• To match psychosocial care carefully and flexibly with oncology treatments.

Recognizing the importance of emotional problems in cancer patients, in 2017 the Hungarian Cancer Society adopted the International Standard of Quality Cancer Care developed by the International Psycho-Oncology Society (IPOS) (57) (https://ipos-society.org/endorsements/organizations):

○ Psychosocial cancer care should be recognized as a universal human right
○ Quality cancer care must integrate the psychosocial domain into routine care

Distress should be measured as the 6th Vital Sign in addition to temperature, blood pressure, pulse, respiratory rate and pain.

#### Psycho-Oncological Assessment and Screening Tools

• Quick screening: Distress Thermometer (measures the degree of distress reported by the patient on a scale of 10; above 4, the patient requires support) and Mitchell’s Emotional Thermometers (58, 59, 63)
• Mood assessment and recording: BDI, Zung, HADS (56, 60)
• Evaluation of anxiety: STAI, HADS (56, 61, 62)
• Problem List: helps to plan individually tailored support by exploring current psychosocial and spiritual difficulties
• Other psychological measuring instruments, depending on the qualification and competence of the psychosocial or mental health professional
• The basic principle of screening is that screened patients should be provided with psychological care and their psychological assessment should be adjusted to their current physical and mental condition
• All newly presenting patients should be included in oncopsychological screening, regardless of whether they had any premorbid psychiatric illness. It is recommended that tests for quick screening are repeated at different stages of the disease (any treatment event, e.g. relapse; or interim periods, e.g., every six months), preferably in conjunction with oncology follow-up (Table 8).

**TABLE 8:** Algorithm for oncopsychological screening.

Distress → short evaluation (e.g. distress thermometer) + list of problems→	Moderate or severe distress, DT = 4 or more	→	Clinical assessment: validated scales, screening tests to measure anxiety/depression (oncologist, nurse, social worker or trained professional) in the following cases:	→Referral,	→Mental healthcare providers Psychiatry/psychology care)
			• high risk patient		
			○ high vulnerability period		
→	Non-relieved physical symptoms (treated according to disease- specific or palliative care guidelines)		○ distress risk factors are present- practical issues	if needed 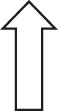	→Social worker and counselling services
			• family issues		
			• spiritual/religious issues		
	Clinically confirmed mild distress or DT <4		• physical problems	Primary oncology care team + available resources	→Spiritual care (pastor)
			• social problems		
			• emotional problems (e.g. anxiety, depression)		
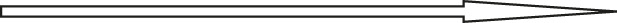					

Source: NCCN Guidelines Version 1. 2020 Distress Management, National Comprehensive Cancer Network (2020) (65).

#### Possibilities for Psychosocial Oncology Care Intervention in Different Phases of the Disease

• Communication of diagnosis: crisis intervention, counselling, supportive therapy, psychodiagnostics, psychosocial screening.
• Initiation of treatment: psychoeducation, reduction of distress, supportive therapies, cognitive and behavioural therapies, couple therapy, life management counselling, “imaginative” therapies.
• Completion of treatment, recovery: verbal and non-verbal psychotherapies.
• Completion of treatment, deteriorating condition: preventive pastoral care, crisis intervention, support for family members, counselling, supportive psychotherapies.
• Death, dying: dignity therapy, crisis intervention, grieving process embedded in psychotherapy, bereavement support groups, self-help bereavement groups.
• An early preventive approach in interventions is important, anticipating the possibility of recurrence and the effectiveness of second- and third-line treatments, supported by statistical data, if necessary.

○ Together with proper communication, this will improve compliance. It will allow for the creation of a long-term therapeutic collaboration plan, the message of which for the patient is that the treating team trusts in their long-term survival and wants to involve the patient in the treatment process.

○ Starting from the communication of the diagnosis, during the step-by-step process of information-treatment-preparation, it is recommended that issues relevant in the longer term, such as possibilities of breast reconstruction, or the issue of having children after breast cancer treatment, be addressed gradually.

#### Professional Conditions for Psychological Support of Cancer Patients

Hungarian National Cancer Control Programme (2006):

• Specialists in the psychosocial treatment of cancer patients (clinical or health psychologist, psychiatrist and/or psychotherapist), working together as members of the oncology team with the oncologist, physiotherapist, dietitian and social worker, should be made available in oncology centres, departments and caregiving services.
• Continuous consultation and documentation between different professions is essential for monitoring changes in the patient’s condition.
• The primary goal is to maintain the best possible quality of life and physical well-being while preserving emotional, social and spiritual well-being.
• Appropriate physical environment and work organization, availability of oncopsychological training/further training.

This is part 2 of a series of 6 publications on the 1st Central-Eastern European Professional Consensus Statements on Breast Cancer covering imaging diagnosis and screening (64), pathological diagnosis (65), surgical treatment (66), systemic treatment (67), radiotherapy (68) of the disease and related follow-up, rehabilitation and psychosocial oncology care issues (present paper).

## Author’s Note

The consensus document contains product placement without the intention of advertising. Each complex molecular test is unique, and although these can be described without indicating their name (for example with the number of genes tested), not everyone will necessarily understand what this refers to. For this reason, and adopting the practice used in some of the source works, the tests are listed under their trade name.
