# Supplementary material for: Breast Cancer Survivorship Programme: Follow-Up, Rehabilitation, Psychosocial Oncology Care. 1st Central-Eastern European Professional Consensus Statement on Breast Cancer
Source: Pathol Oncol Res. 2022 Jun 2;28:1610391. doi: 10.3389/pore.2022.1610391 (PMC9200958; doi:10.3389/pore.2022.1610391)
Supplement: Supplementary file 1 [file Table1.DOCX]

| **Country** | **Name** | **Institute** |
| --- | --- | --- |
| **Armenia** | Artur Avetisyan, MD, PhD | Deputy director of the National Center of Oncology/NCO |
|  | Metaksia Mkrtchyan, MD | Surgeon at the Women`s health clinic at the NCO |
|  | Anna Tadevosyan, MD, PhD | Medical oncologist of the Chemotherapy department |
|  | Karine Soghomonyan, MD | Medical oncologist of the Chemotherapy department |
|  | Armen Mkhitaryan, MD, PhD | “HistoGen” Practical-scientific Center of Pathology, Department of Pathology of the Yerevan State Medical University/YSMU |
|  | Hasmik Davtyan, MD, PhD | Radiologist at the NCO |
|  | Nune Mashuryan, MD | Head of Breast Health Imaging Service at “Astghik” Medical Center |
|  | Tatul Saghatelyan, MD, PhD | Head of the Radiotherapy department at the NCO |
|  | Anna Baghdasaryan | Onco-psychologist at the NCO |
|  | Anahit Petrosyan, MD | Rehabilitation physician, Head of “Lymph Reha-Center” |
|  | Hripsime Martirosyan | Director of HENARAN cancer patient organization |
| **Azerbaijan** | Dr. Javid Garibov | Azerbaijan Republic Ministry of Health - National Center of Oncology |
|  | Prof. Jamil Aliyev | Azerbaijan Republic Ministry of Health - National Center of Oncology |
|  | Dr. Tahir Najafov | Azerbaijan Republic Ministry of Health - National Center of Oncology |
|  | Dr. Lamiyya Majidova | Azerbaijan Republic Ministry of Health - National Center of Oncology |
|  | Dr. Tunzala Mammadova | Azerbaijan Republic Ministry of Health - National Center of Oncology |
| **Belarus** | Krasny S.A MD DsC | Vice director of N.N.Alexandrov National cancer centre of Belarus |
|  | Khorau A.O MD PhD | Head of the reconstructive surgery and breast cancer laboratory of N.N.Alexandrov National cancer centre of Belarus |
|  | Mikulich D.V. | Researcher of the reconstructive surgery and breast cancer laboratory of N.N.Alexandrov National cancer centre of Belarus |
| **Bosnia and Herzegovina** | Prof Dr Semir Beslija | Head of Oncology Clinic Sarajevo - Clinical Center University of Sarajevo |
|  | Prof dr Timur Ceric | Oncology Clinic Sarajevo - Clinical Center University of Sarajevo |
|  | Prof dr Zdenka Gojkovic | Head of the UBKC Banja Luka Oncology Clinic |
| **Bulgaria** | Nataliya Chilingirova, MD, PhD | Science and Research Institute Medical University Pleven Head of Medical Oncology Clinic Center of Excellence, Heart and Brain Hospital Pleven |
| **Georgia** | Prof. Rema Gvamichava MD, PhD | Director - Radiacion  Medical Center, Head - Department of Oncology, Tblisi State Medical University, President - Georgian Society of Clinical Oncology |
|  | Professor George Burkadze, MD, PhD, IFCAP | Head of the Department of the Molecular Pathology of Tbilisi State Medical University, President of Association of Pathologists and Cytopathologists of Georgia |
|  | Teona Muzashvili, MD, PhD | Head of the branch of immunohistochemistry at Tblisi State Medical University Pathology Study-Scientific and Diagnostic Laboratory, Pathologist |
|  | Nino Sharikadze MD | Oncohematologis/Medical Oncologist. Head of Mardaleishvili Medical Centre |
|  | Dr. George Orkodashvili  M.D., Ph.D | Radiation Oncologist Radiation Medicine Centre (Tbilisi, Georgia) |
|  | I. Kokhreidze MD., PhD | Tblisi State Medical University, Associated Professor of Oncology Department |
| **Hungary** | Prof. Miklós Kásler MD, PhD, DSc, drhc, FRCS | Minister of Human Resources, Government of Hungary |
|  | Gábor Forrai MD, PhD | GÉ-RAD Kft., DMC, Budapest |
|  | Eszter Kovács MD | GÉ-RAD Kft., Duna Medical Center, Budapest |
|  | Éva Ambrózay MD | Mamma Egészségügyi Zrt., Budapest, Kecskemét, Szekszárd, Szolnok |
|  | Miklós Barta MD | Royal Cornwall Hospital, Truro, UK |
|  | Prof. Katalin Borbély MD, PhD, med habil, DSc | National Institute of Oncology, Ministry of Human Capacities,Budapest |
|  | Zsolt Lengyel MD, PhD | Hamad Medical Corporation, Doha, Qatar |
|  | Katalin Ormándi MD | University of Szeged Department of Radiology, Szeged |
|  | Zoltán Péntek MD, PhD | Mamma Egészségügyi Zrt., Budapest, Kecskemét, Szekszárd, Szolnok |
|  | Tünde Tasnádi MD | Dr Réthy Pál Member Hospital of Békés County Central Hospital, Békéscsaba |
|  | Éva Sebő MD | University of Debrecen, Kenézy Gyula University Hospital, Debrecen |
|  | Prof. Gábor Cserni MD, PhD, DSc | Department of Pathology, Bács-Kiskun County Teaching Hospital, Kecskemét  Institute of Pathology, University of Szeged, Albert Szent-Györgyi Clinical Center, Szeged |
|  | Mónika Francz MD | Department of Pathology, Szabolcs-Szatmár-Bereg County Hospitals and University Teaching Hospital, “Jósa András” Teaching Hospital, Nyíregyháza |
|  | Balázs Járay MD, PhD | Medserv Kft., Budapest |
|  | Endre Kálmán MD | Institute of Pathology, University of Pécs, Pécs |
|  | Ilona Kovács MD | Department of Pathology, University of Debrecen, “Kenézy Gyula” University Hospital, Debrecen |
|  | Tibor Krenács MD | Department of Pathology and Experimental Cancer Research, Semmelweis University, Budapest, Hungary |
|  | Erika Tóth MD, PhD | Department of Pathology, National Institute of Oncology, Budapest |
|  | Nóra Udvarhelyi MD | Department of Pathology, National Institute of Oncology, Budapest |
|  | László Vass MD, PhD | Department of Pathology, Pest County “Flór Ferenc” University Teaching Hospital, Kistarcsa |
|  | András Vörös MD | Institute of Pathology, University of Szeged, Szeged |
|  | Prof. Janina Kulka MD, PhD, DSc | Department of Pathology, Forensic and Insurance Medicine, Semmelweis University, Budapest |
|  | Zoltán Mátrai MD, PhD, med habil | Dept. of Breast and Sarcoma Surgery, National Institute of Oncology, Budapest |
|  | Péter Kelemen MD, PhD | Dept. of Breast and Sarcoma Surgery, National Institute of Oncology, Budapest |
|  | Csaba Kósa MD, PhD | University of Debrecen, Department of Surgery, Debrecen |
|  | Róbert Maráz MD, PhD | Bács-Kiskun County Teaching Hospital, Kecskemét |
|  | Attila Paszt MD, PhD | SZTE ÁOK, University of Szeged - Faculty of Medicine, Department of Surgery, Szeged |
|  | Gábor Pavlovics MD | University of Pécs, Department of Surgery, Pécs |
|  | Ákos Sávolt MD, PhD | Dept. of Breast and Sarcoma Surgery, National Institute of Oncology, Budapest |
|  | Zsolt Simonka MD, PhD | SZTE ÁOK, University of Szeged - Faculty of Medicine, Department of Surgery, Szeged |
|  | Dezső Tóth MD, PhD | University of Debrecen, Department of Surgery, Debrecen |
|  | Prof. György Lázár MD, PhD, DSc | SZTE ÁOK, University of Szeged - Faculty of Medicine, Department of Surgery, Szeged |
|  | Gábor Rubovszky MD, PhD | National Institute of Oncology, Chest and Abdominal Tumours Chemotherapy “B” and Department of Clinical Pharmacology, Budapest |
|  | Judit Kocsis MD, PhD, med habil | Bács-Kiskun County Teaching Hospital, Center of Oncoradiology, Kecskemét |
|  | Katalin Boér MD, PhD | Szent Margit Hospital, Department of Oncology, Budapest |
|  | Prof. Magdolna Dank MD, PhD | Semmelweis University, Oncology Centre, Budapest |
|  | Prof. Zsuzsanna Kahán MD, PhD, DSc | Department of Oncotherapy, University of Szeged, Szeged, Hungary. |
|  | Erika Kövér MD, PhD | University of Pécs, Faculty of Medicine, Institute of Oncotherapy, Pécs |
|  | Károly Máhr MD, PhD | Szent Rafael Hospital of Zala County, Department of Oncology, Zalaegerszeg |
|  | Béla Pikó MD, PhD | Pándy Kálmán Hospital of Békés County Council, County Oncology Centre), Gyula |
|  | Zsolt Horváth MD, PhD | Bács-Kiskun County Teaching Hospital, Center of Oncoradiology, Kecskemét |
|  | Prof. Csaba Polgár MD, PhD, med habil, DSc | Centre of Radiotherapy, National Institute of Oncology, Budapest, Department of Oncology, Semmelweis University, Budapest |
|  | András Csejtei MD, PhD | Department of Oncoradiology, Markusovszky University Teaching Hospital, Szombathely |
|  | Gabriella Gábor MD | Oncoradiology Centre Bács-Kiskun County Hospital, Kecskemét |
|  | László Landherr MD, PhD | Municipial Oncoradiology Centre, Uzsoki Street Hospital, Budapest |
|  | László Mangel MD, PhD, med habil | Oncotherapy Institute, University of Pécs, Pécs |
|  | Árpád Mayer MD, PhD, DSc | Department of Oncoradiology, Markusovszky University Teaching Hospital, Szombathely |
|  | János Fodor MD, PhD, med habil, DSc | Centre of Radiotherapy, National Institute of Oncology, Budapest |
|  | István Szántó MD, PhD | St.George’s General Teaching Hospital, Fejér County, Székesfehérvár |
|  | Rita Dudás MD | Department of Oncotherapy, University of Szeged, Szeged |
|  | Zsuzsanna Kapitány | Department of Physiotherapy, Semmelweis University, Budapest |
|  | Mária Molnár | Oncoradiology Centre, Bács-Kiskun County Hospital, Kecskemét |
|  | Zsuzsa Koncz | Institute of Behavioural Sciences, Semmelweis University, Budapest |
|  | Mónika Mailáth | Institute of Oncology, University of Debrecen, Debrecen |
| **Kazakhstan** | Dilyara Kaidarova MD, Prof. | Chairman of the Board Kazakh Institute of Oncology and Radiology |
|  | Abdrakmanov Ramil , MD, PhD | Head of Medical oncology division, Kazakh Institute of oncology and radiology |
|  | Smagulova Kaldygul, MD, PhD | Head of outpatient chemotherapy department, Kazakh Institute of oncology and radiology |
|  | Abdrakhmanova Aliya, MD | Head of Breast Cancer Center, Kazakh Institute of Oncology and radiology |
|  | Omarbayeva Nazgul MD | Breast surgeon, Breast Cancer Center, Kazakh Institute of oncology and radiology |
|  | Savkhatova Akmaral MD PhD | Radiation oncologist, Head of Radiation therapy division |
|  | Zhandos Amankulov PhD | Head of Radiology Division, Kazakh Institute of Oncology and Radiology |
|  | Ainakulova Akmaral MD PhD | Radiologist, Kazakh Institute of Oncology and Radiology |
|  | Satpaeva Elvira MD PhD | Head of pathology division, Kazakh Institute of oncology and radiology |
|  | Anarbayev Nurgali MD PhD | Pathologist, Kazakh Institute of oncology and radiology |
|  | Omarbayeva Nazgul MD PhD | Breast surgeon, Breast Cancer Center, Kazakh Institute of oncology and radiology |
| **Montenegro** | Prof. dr Vladimir Todorovic | Institute for Oncology in Podgorica, Montenegro |
| **Poland** | Prof. Agnieszka Kolacinska-Wow | Dept. of Head and Neck Cancer Surgery, Medical University of Lodz, Poland, Dept. of Surgical Oncology, Cancer Center, Lodz, Poland |
|  | Prof. Jerzy Jankau  (dr hab n med) | Plastic Surgery Department, Medical University of Gdańsk/University Hospitals, Poland |
|  | Prof. Dawid Murawa | Clinic of Surgical Oncology, Poznan University of Medical Sciences, Poznan, Poland; General and Oncological Surgery Clinic, Karol Marcinkowski University Hospital, Zielona Gora, Poland |
|  | Dr Piotr Pluta | Department of Surgical Oncology and Breast Diseases Polish Mother’s Memorial Hospital – Research Institute in Lodz, Poland |
|  | Dr Mateusz Wichtowski | Centre for Cancer Prevention and Epidemiology |
|  | Slawomir Ciesla | General and Oncological Surgery Clinic, Karol Marcinkowski University Hospital, Zielona Gora, Poland |
| **Romania** | Alexandru Blidaru MD PhD | Department of Surgery, University of Medicine and Pharmacy “Carol Davila” Bucharest Head of the Surgical and Gynecological Oncology II Department – “Prof. Dr. Alexandru Trestioreanu” Institute of Oncology Bucharest President of the Romanian Society of Breast Surgery and Oncology |
|  | Cristian Ioan Bordea MD PhD | Assistant prof. of Surgical Oncology, MD, University of Medicine and Pharmacy “Carol Davila” Bucharest Senior Surgeon, Consultant Surgical Oncology “Prof. Dr. Alexandru Trestioreanu” Institute of Oncology Bucharest |
|  | Dan Corneliu Jinga MD, PhD | Head of Department of Medical Oncology, Neolife Bucharest Hospital |
|  | Liviu Stoleru MD | Senior Consultant Radiotherapy, Neolife Bucharest Hospital |
|  | Cristian Radu Jecan MD, PhD | Professor of Plastic, Aesthetic and Reconstructive Microsurgery, University of Medicine and Pharmacy “Carol Davila” Bucharest Head of Department Plastic, Aesthetic and Reconstructive Microsurgery, “Agrippa Ionescu” Hospital, Bucharest President of the Romanian Association of Plastic Surgery |
|  | Mirela Gherghe MD, PhD | Lecturer University of Medicine and Pharmacy “Carol Davila” Bucharest, Chief of Nuclear Medicine Discipline Senior Consultant in Nuclear Medicine, Head of Department Nuclear Medicine “Prof. Dr. Alexandru Trestioreanu” Institute of Oncology Bucharest |
|  | Mihaela Alexandra Radu, MD | Assistant Professor of Surgical Oncology, University of Medicine and Pharmacy “Carol Davila” Bucharest Vice-president of the Romanian Society of Breast Surgery and Oncology Obstetrics and Gynecology - Profmedica Bucharest, Medicover Hospital |
|  | Aniela Roxana Noditi, MD | Vice president of the Romanian Society of Breast Surgery and Oncology General Surgery - Profmedica Bucharest, “Prof. Dr. Alexandru Trestioreanu” Institute of Oncology Bucharest |
| **Russian Federation** | Prof. Andrey Kaprin | Director General, National Medical Research Radiological Center (NMRRC) of the Ministry of Health of the Russian Federation Chief oncologist of the Russian Federation Russian Academy of Science full member Moscow, Russian Federation |
|  | Prof. ZYKIRYAHOJAEV Aziz, MD | Head of the Department of Oncology and Reconstructive Plastic Surgery of the Breast and Skin P. Hertsen Moscow Oncology Research Institute, the branch of the National Medical Research Radiological Centre of the Ministry of Health of the Russian Federation |
|  | Dr.ONOFRIYCHUK Irina, PhD | Oncologist, Department of Oncology and Reconstructive Plastic Surgery of the Breast and Skin P. Hertsen Moscow Oncology Research Institute, the branch of the National Medical Research Radiological Centre of the Ministry of Health of the Russian Federation |
|  | Dr.FEDENKO Aleksandr, MD | Head of the Department of Drug Treatment of Tumors P. Hertsen Moscow Oncology Research Institute, the branch of the National Medical Research Radiological Centre of the Ministry of Health of the Russian Federation |
|  | Dr.BOLOTINA Larisa, MD | Head of the Chemotherapy Department P. Hertsen Moscow Oncology Research Institute, the branch of the National Medical Research Radiological Centre of the Ministry of Health of the Russian Federation |
|  | Prof.KHMELEVSKY Yeugeniy, MD | Head of the Radiotherapy Department P. Hertsen Moscow Oncology Research Institute, the branch of the National Medical Research Radiological Centre of the Ministry of Health of the Russian Federation |
|  | Dr.MEDVEDEV Serguei, PhD | Senior Researcher, oncologist, radiotherapist P. Hertsen Moscow Oncology Research Institute, the branch of the National Medical Research Radiological Centre of the Ministry of Health of the Russian Federation |
|  | Alexander V. Petrovsky MD. PhD | Deputy Director  Head of Breast Surgery Department  N.N. Blokhin National Cancer Research Center  Associate professor in oncology  I.M. Sechenov Moscow Medical State University |
|  | Petr Krivorotko MD PhD. D.Sc. | Assistant-director of the N.N. Petrov NMRC |
|  | Julianna Shatova | Head of the breast cancer dpt. of the Rostov NMRC |
|  | Dr Arthur Ismagilov | Head of the plastic surgery dpt. of the Kazan medical Academy |
|  | Alexander Bessonov MD PhD | Head of Breast Cancer Dept. of the LOKOD, assistant-professor of the N.N. Petrov NMRC |
|  | Sergey Novikov MD PhD | Head of the Radiotherapy Dept. of the N.N. Petrov NMRC |
|  | Anna Artemyeva | Head of the Pathology Dept. of the N.N. Petrov NMRC |
| **Serbia** | prof dr Dragana Djilas | Consilium Polyclinic |
|  | prof dr Tatjana Ivković Kapicl | Institute of Oncology of Vojvodina, Department of Pathological-Anatomical and Laboratory Diagnostics |
|  | Ana Krivokuća | Head of Genetic counseling department, Institute for Oncology and Radiology of Serbia |
|  | prof dr Ferenc Vicko | State Secretary of Ministry of Health |
|  | prof dr Ivana Božović Spasojević | Head of the Daily Chemotherapy Hospital at the Institute for Oncology and Radiology, National Cancer Research Centre in Belgrade, Republic of Serbia |
|  | ass dr sci med Olivera Ivanov | Institute of Oncology of Vojvodina, Clinic for Radiological Therapy |
|  | prof dr Svetlana Petrović Popović | Institute of Oncology of Vojvodina, Department of Physical Medicine and Rehabilitation |
| **Slovakia** | assoc. prof. MUDr. Karol Kajo, PhD., | Dept. of Pathology of St. Elisabeth Cancer Institute, Bratislava |
|  | MUDr. Katarína Kajová Macháleková, PhD | Dept. of Pathology of St. Elisabeth Cancer Institute, Bratislava |
|  | MUDr. Lucia Vanovnčanová, PhD | Medical faculty of Comenius University, Bratislava, and Dept. of Radiology of St. Elisabeth Cancer Institute |
|  | assoc. prof. MUDr. Martin Sabol, PhD., MUDr | Dept. of Surgical Oncology of St. Elisabeth Cancer Institute and Medical Faculty of Comenius University |
|  | MUDr. Daniel Dyttert, PhD., | Dept. of Surgical Oncology of St. Elisabeth Cancer Institute and Medical Faculty of Comenius University |
|  | MUDr. Peter Chvalný PhD | Dept. of Surgical Oncology of St. Elisabeth Cancer Institute and Medical Faculty of Comenius University |
|  | MUDr. Martin Chorvát, PhD. MHA | Department of Radiation Oncology of Slovak Medical University in Bratislava and St. Elisabeth Cancer Institute |
|  | MUDr. Andrea Ligačová, | Department of Radiation Oncology of Slovak Medical University in Bratislava and St. Elisabeth Cancer Institute |
|  | MUDr. Pavol Lukačko | Department of Radiation Oncology of Slovak Medical University in Bratislava and St. Elisabeth Cancer Institute |
|  | MUDr. Bela Mriňáková, PhD., MHA | 1st Oncology Department of Medical Faculty of Comenius University, Bratislava, and Medical Oncology department of St. Elisabeth Cancer Institute |
|  | MUDr. Bibiana Vertáková Krakovská, PhD | 1st Oncology Department of Medical Faculty of Comenius University, Bratislava, and Medical Oncology department of St. Elisabeth Cancer Institute |
|  | prof. MUDr. Stanislav Špánik, CSc | President of the Slovak Oncology Society |
| **Tajikistan** | Prof. Dr Zafar Huseynzoda | Director of the Russian Cancer Research Center |
| **Ukraine** | Zhygulin Andrii MD | LISOD Hospital of Israeli Oncology, Kyiv |
|  | Ponomarova Olga | Kyiv Municipal Clinical Oncological Center, Shupyk National Medical Academy of Postgraduate Education, Kyiv |
|  | Lyalkin Sergii MD. PhD | Senior researcher, Department of Chemotherapy of solid tumors, National Cancer institute, Kyiv. Member of ESMO, |
|  | Kovalyov Olexiy MD. PhD. Prof | Zaporizhzhia Academy of Postgraduate Education, Zaporizhzhia |
|  | Shparyk Yaroslaw MD, Phd, Ass Prof. | Lviv State Regional Oncological Center; Lviv Medical University; Lviv |
|  | Zavizion Victor MD, PhD, Ass Prof. | Dnipro Medical University, Dnipro |
|  | Ryspayeva Dinara MD, PhD, Ass Prof. | LISOD Hospital of Israeli Oncology, Kyiv |
|  | Natalya Lisovska MD. PhD | Medical Center Clinic of Spizhenko, Kyiv |
|  | Zotov Olexiy MD, PhD. | Bohomolets National Medical University, Kyiv National Institute of Surgery and Transplantology of the National Academy of Sciences of Ukraine; Kyiv. |
|  | Smolanka Ivan | Manufactura Clinic |
|  | Lyashenko Andrii MD, PhD, | National Cancer Institute, Kyiv |
|  | Savenkov Oleg MD | Medical center “Garvis”, Dnipro Dnipro State oncological hospital |
|  | Pominchuk Denys MD, PhD. | Medical center “Verum expert”, Kyiv |
|  | Cheshuk Valerii PhD. Prof. | Kyiv Municipal Clinical Oncological Center; Research Center for Continuous Professional Development of the Bohomolets National Medical University, Kyiv |
|  | Koshyk Olena MD. | CSD Medical Laboratory,  Kyiv |
|  | Shapochka Dmytro | Preci laboratory, Kyiv |
|  | Bozhok Ievgen MD, PhD. | SI Institute of Nuclear Medicine and Radiology of National Academy of Medical Sciences of Ukraine |
|  | Gurando Andrii MD. | SI Institute of Nuclear Medicine and Diagnostic Radiology of the National Academy of Medical Science of Ukraine, Kyiv |
|  | Vinnytska Daria | LISOD Hospital of Israeli Oncology, Kyiv |
|  | Novikov Mykola MD., PhD. | LISOD Hospital of Israeli Oncology, Kyiv |
|  | Musaiev Burkhan | Clinic "M24", Kyiv |
|  | Bernstein Zvi MD. | LISOD Hospital of Israeli Oncology, Kyiv |
|  | Serogina Nataliia MD. | Ukrainian Center of Tomotherapy, TOMOCLINIC, Kropyvnytsky |
|  | Buchynskyi Sergii PRM doctor | Department of rehabilitation and physiotherapy of the KNE CDC Shevchenko district, Kyiv. |
|  | Koval Svitlana | LISOD Hospital of Israeli Oncology, Kyiv |
|  | Diakovska Olha | LISOD Hospital of Israeli Oncology, Kyiv |
|  | Rybak Natalia | LISOD Hospital of Israeli Oncology, Kyiv |
|  | Slabinska Iya | LISOD Hospital of Israeli Oncology, Kyiv |
|  | Dziuba Janina | Kyiv, Private practice. |
