# Supplementary material for: Breast Cancer Survivorship Programme: Follow-Up, Rehabilitation, Psychosocial Oncology Care. 1st Central-Eastern European Professional Consensus Statement on Breast Cancer
Source: Pathol Oncol Res. 2022 Jun 2;28:1610391. doi: 10.3389/pore.2022.1610391 (PMC9200958; doi:10.3389/pore.2022.1610391)
Supplement: Supplementary file 2 [file DataSheet1.pdf]

APPENDIX 1

Forms of social security allowances in Hungary

Sickness benefit: the entitlement to sickness benefits has three basic conditions: an existing social insurance status, contributions paid until the time of incapacity to work, and incapacity to work established and certified by a physician. It can be granted for a maximum period of one year, but health insurance status must be valid throughout this period. The amount of sickness benefit is 60% of the income representing the contribution base, but it may not exceed the one-thirtieth part of the double of the minimum wage. In 2020, this amount may be HUF 10,728 per day.

Care for people with disabilities: the assessment of altered work capacity is regulated by a complex qualification system (Decree No. 7/2012. II.14. of the NEFMI – Minister of National Resources, Hungary). With this qualification system, statuses of health, employment and possible social rehabilitation are assessed. During this complex qualification process the rehabilitation authority will establish whether the person with an altered working capacity can be rehabilitated or rehabilitation is not recommended. Accordingly, the patient is paid either a rehabilitation benefit or disability benefit, the amount of which depends on the degree of health impairment (http://emmiugyfelszolgalat.gov.hu/szocialis/megvaltozott, http://www.kormanyhivatal.hu/download) (*Table 1a*).

Social benefits in case of social need (the competence of the local government and based on need)

Benefits for patients of working age: a form of benefit that can be provided to individuals disadvantaged in the labour market and their families. One form is the health impairment benefit, which is a regular allowance provided by the district office to people who are not capable of working and have a health impairment of at least 50%.

A regular carer's allowance can be granted for a person caring for a chronically ill relative at their home. The GP will issue a certificate about the long-term illness or severely disabled condition, and the application will be judged by the district office.

Local municipality support: Regular or one-time local municipality support may be established for persons in a life situation that jeopardizes their subsistence and for persons with temporary or permanent living problems. In particular, it may be granted to persons bearing regular expenses related to housing, or as subsidies for medicines, occasional extra expenses or persons accumulating arrears. As a social benefit in kind, the district office may provide the persons in need with access to health care services (if they do not have valid social insurance) and normative free public health services. A person who receives disability benefits and whose state of health does not exceed 30% on the basis of a complex qualification by the rehabilitation authority has access to free public health services on the basis of subjective right. An application for free public health services can be submitted to the district office competent according to the patient's place of residence and to the government office (http://emmiugyfelszolgalat.gov.hu/szocialis/szocialis-raszorultsag).

Individual social services

If the patient's/client's family is unable to take care of the sick relative since they are worried about losing their jobs, or if the burden of caring for the patient exceeds the family's capabilities, home care services may assist in caring for patients/clients in need of specialist nursing. Organizing basic services helps those in need to maintain independent living in their

own home and living environment. Patients discharged home but in need of support can be provided with home help, care, meals, an alert system, and family assistance. For homeless patients/clients, temporary accommodation for homeless people, daytime warming and night shelter provide solutions in a crisis situation. Care for individuals who cannot care for themselves at all (or only with continuous assistance) is provided in nursing and care institutions, provided that their care needs cannot be met in any other way. Information provided by social workers, and communication with supporting organizations and institutional systems (e.g. family caregivers, child welfare services, GP, local government, social security institution, patient rights representatives) help to solve the patients'/clients' social problems.

Table 1a: Disability benefit and rehabilitation benefit allowances (Hungary, 2020)
In accordance with Act CXCI of 2011 (Mmtv.) on the benefits for persons with reduced capacity to work and amending certain acts (Mmtv.). Valid basic amount: HUF 104,405 (01/01/2020) (33, 34)

**Disability benefits**			
	Amount of benefit	Minimum amount	Maximum amount
Health status between 51 and 60% (category B2): The patient's working capacity can be restored with rehabilitation; however, the patient cannot be employed due to other circumstances specified in a separate regulation, their occupational rehabilitation is not recommended	40% of the average monthly income	HUF 31,325 (30% of the basic amount)	HUF 46,985 (45% of the basic amount)
Health status between 31 and 50% (category C2): Requires permanent rehabilitation; however, the patient cannot be employed due to other circumstances specified in a separate regulation, their occupational rehabilitation is not recommended	60% of the average monthly income	HUF 46,985 (45% of the basic amount)	HUF 156,610 (150% of the basic amount)
Health status between 1 and 30% (category D): The patient may only be employed with ongoing support	65% of the average monthly income	HUF 52,205 (50% of the basic amount)	HUF 156,610 (150% of the basic amount)
Health status between 1 and 30% (category E): In case of total or partial loss of self-sufficiency	70% of the average monthly income	HUF 57,425 (55% of the basic amount)	HUF 156,610 (150% of the basic amount)
**Rehabilitation benefits**			
	Amount of benefit	Minimum amount	Maximum amount
Health status between 51 and 60% (category B1): The patient's employability can be restored with rehabilitation	35% of the average monthly income	HUF 31,325 (30% of the basic amount)	HUF 41,765 (40% of the basic amount)
Health status between 31 and 50% (category C1): Requires long-term occupational rehabilitation	45% of the average monthly income	HUF 41,765 (40% of the basic amount)	HUF 52,205 (50% of the basic amount)
